# ERK and mTORC1 Inhibitors Enhance the Anti-Cancer Capacity of the Octpep-1 Venom-Derived Peptide in Melanoma BRAF(V600E) Mutations

**DOI:** 10.3390/toxins13020146

**Published:** 2021-02-14

**Authors:** Javier Moral-Sanz, Manuel A. Fernandez-Rojo, Jeremy Potriquet, Pamela Mukhopadhyay, Andreas Brust, Patrick Wilhelm, Taylor B. Smallwood, Richard J. Clark, Bryan G. Fry, Paul F. Alewood, Nicola Waddell, John J. Miles, Jason P. Mulvenna, Maria P. Ikonomopoulou

**Affiliations:** 1 Translational Venomics Group, Madrid Institute for Advanced Studies in Food, E28049 Madrid, Spain; javier.moral@imdea.org; 2 Hepatic Regenerative Medicine Group, Madrid Institute for Advanced Studies in Food, E28049 Madrid, Spain; manuel.fernandez@imdea.org; 3 Diamantina Institute, The University of Queensland, Brisbane, QLD 4072, Australia; 4 Department of Cell and Molecular Biology, QIMR Berghofer Medical Research Institute, Brisbane, QLD 4006, Australia; 5 Infectious Diseases Program, QIMR Berghofer Medical Research Institute, Brisbane, QLD 4006, Australia; Jeremy.Potriquet@sciex.com (J.P.); jason.mulvenna@gmail.com (J.P.M.); 6 AB SCIEX, 2 Gilda Court, Mulgrave, Melbourne, VIC 3170, Australia; 7 Department of Genetics and Computational Biology, QIMR Berghofer Medical Research Institute, Brisbane, QLD 4006, Australia; Pamela.Mukhopadhyay@qimrberghofer.edu.au (P.M.); Nic.Waddell@qimrberghofer.edu.au (N.W.); 8 Institute for Molecular Bioscience, The University of Queensland, Brisbane, QLD 4072, Australia; andreas.brust@iinet.net.au (A.B.); patrickwilhelm@fastmail.com (P.W.); richard.clark@uq.edu.au (R.J.C.); p.alewood@imb.uq.edu.au (P.F.A.); 9 School of Biomedical Sciences, The University of Queensland, Brisbane, QLD 4072, Australia; t.smallwood@uq.edu.au; 10 School of Biological Sciences, The University of Queensland, Brisbane, QLD 4072, Australia; bgfry@uq.edu.au; 11 Department of Immunology, QIMR Berghofer Medical Research Institute, Brisbane, QLD 4006, Australia; john.miles@jcu.edu.au; 12 The Australian Institute of Tropical Health and Medicine (AITHM), James Cook University, Cairns, QLD 4811, Australia; 13 Centre for Molecular Therapeutics, James Cook University, Cairns, QLD 4811, Australia; 14 Centre for Tropical Bioinformatics and Molecular Biology, James Cook University, Cairns, QLD 4811, Australia

**Keywords:** melanoma BRAF mutation, cancer, combination-therapies, venom-peptide, octopus-peptide

## Abstract

Melanoma is the main cause of skin cancer deaths, with special emphasis in those cases carrying BRAF mutations that trigger the mitogen-activated protein kinases (MAPK) signaling and unrestrained cell proliferation in the absence of mitogens. Current therapies targeting MAPK are hindered by drug resistance and relapse that rely on metabolic rewiring and Akt activation. To identify new drug candidates against melanoma, we investigated the molecular mechanism of action of the *Octopus Kaurna*-derived peptide, Octpep-1, in human BRAF(V600E) melanoma cells using proteomics and RNAseq coupled with metabolic analysis. Fluorescence microscopy verified that Octpep-1 tagged with fluorescein enters MM96L and NFF cells and distributes preferentially in the perinuclear area of MM96L cells. Proteomics and RNAseq revealed that Octpep-1 targets PI3K/AKT/mTOR signaling in MM96L cells. In addition, Octpep-1 combined with rapamycin (mTORC1 inhibitor) or LY3214996 (ERK1/2 inhibitor) augmented the cytotoxicity against BRAF(V600E) melanoma cells in comparison with the inhibitors or Octpep-1 alone. Octpep-1-treated MM96L cells displayed reduced glycolysis and mitochondrial respiration when combined with LY3214996. Altogether these data support Octpep-1 as an optimal candidate in combination therapies for melanoma BRAF(V600E) mutations.

## 1. Introduction

Melanoma is the most prominent and lethal skin cancer. The main melanoma mutation is the substitution of a valine to glutamine in codon 600 (V600E) of the serine-threonine kinase BRAF(V600E) which accounts for ~50% of cases [1]. Melanoma is treatable at early stages by surgical removal. However, advanced or metastatic melanoma becomes lethal.

Breakthroughs have occurred in the treatment of progressed melanoma based on comprehension of the oncogenic signaling, genetic alterations and the immunobiology of this cancer. BRAF inhibitor monotherapy (BRAFi) with the approval of vemurafenib and dabrafenib by the US Food and Drug Administration (FDA) constituted pivotal treatments against melanoma. BRAFi monotherapy has proved to be superior to MEK inhibitor (MEKi) monotherapy, with the latter linked to a narrower therapeutic window [2]. Unfortunately, in both treatments, patients acquire resistance and eventually relapse [3]. Drivers of acquired resistance and toxicity of the BRAFi are diverse and include mechanisms leading to paradoxical activation of the mitogen-activated protein kinase (MAPK) pathway [3,4]. Interestingly, combining inhibitors of MEK and mutant BRAF kinase delays MAPK-driven acquired resistance and prolongs the duration of responses, achieving higher rate of tumor responses and decreases associated toxicities derived from the paradoxical MAPK pathway activation [3]. Hence, in advanced BRAF(V600E) melanoma, the standard of care is the combination of BRAF and MEK inhibitors (dabrafenib and trametinib) [3]. Moreover, the most recently explored option is the triple combination therapy of BRAF and MEK inhibitors with immunotherapy in patients with BRAF(V600E) metastatic melanoma. The combination of BRAFi (dabrafenib) and MEKi (trametinib) with pmel-1 adoptive cell transfer (ACT) causes tumor regression, increases T cell infiltration into tumors and importantly improves in vivo cytotoxicity [5]. Nevertheless, targeted therapies have been associated with rapid deterioration and death following development of secondary resistance. Hence, there is still the unmet medical need for more efficient targeted approaches against advanced or metastatic BRAF(V600E) melanoma.

In this study we investigated the antitumoral mechanism of action of an octopus-derived peptide (Octpep-1) in BRAF(V600E) melanoma cells. Octpep-1 is a modified version of the previously identified peptide (OCT-TK-III) by the transcriptomics analysis of the *Octopus Kaurna* [6]. We identified the most affected signaling pathways via proteomics and RNAseq analysis and validated the importance of AKT pathway by Western blotting. Finally, we discovered that the combination of Octpep-1 with rapamycin or ERK1/2 inhibitor acts synergistically to potentiate cytotoxicity. These results were coupled with seahorse flux technology to unravel metabolic alterations associated with augmented BRAF(V600E) melanoma cell death caused by the Octpep-1/ERKi combinatory treatment. Altogether, we highlight the therapeutic potential of Octpept-1 in melanoma patients.

## 2. Results

### 2.1. Fluorescent Microscopy

To decipher the mode of action of Octpep-1 we used light and fluorescence microscopy in MM96L melanoma cells exposed to the fluoro-tagged peptide. The delivery of functional proteins to cells and the use of cell-permeable peptides have received more attention in the last decade given the potential for therapeutic intervention. Many small peptides traverse the plasma membrane of cells in a concentration-dependent manner and by processes that can be either dependent or independent of receptors [7,8]. Once translocated, these peptides may exert a particular biological function that can be cell-dependent within the penetrated tissue [9,10,11]. Of particular interest are cell penetrating peptides (CPPs), which are small (i.e., less than 30 amino acids) can enter the cells, and have low cytotoxicity [12,13]. In addition, their coupling to various carriers enables their efficient internalization into cells [13,14,15]. Thus, the discovery of CPPs and their ability to transport cargoes into cells has revolutionized macromolecule-and nanoparticle-based cancer treatments [12,13,15].

**Figure 1:**
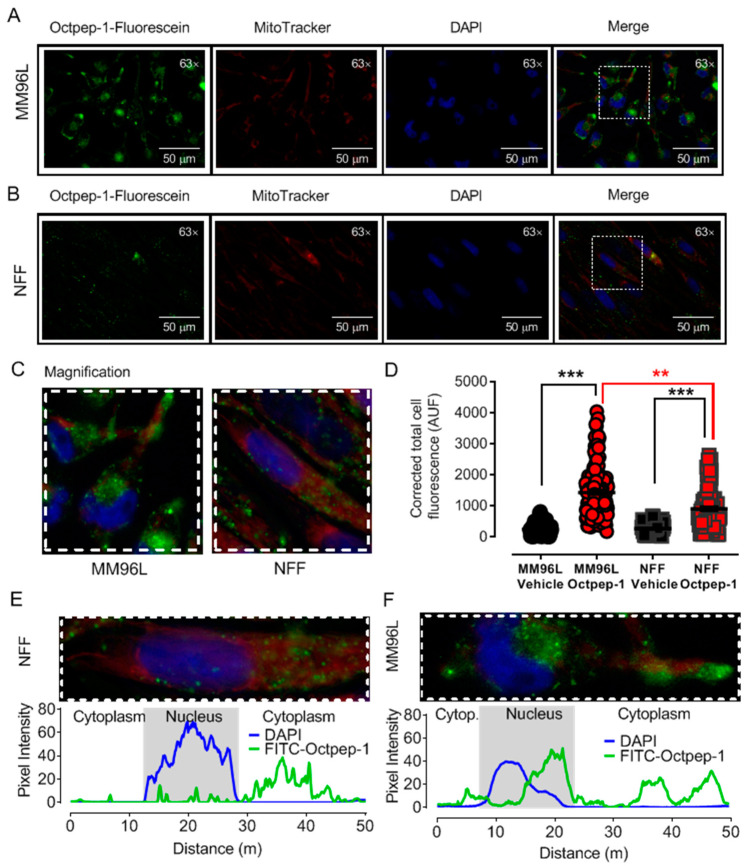
Octpep-1 internalization and intracellular distribution is increased in MM96L as compared to neonatal foreskin fibroblasts (NFF). (**A**) Representative images of MM96L and (**B**) NFF cells treated with Octpep-1-fluorescein and co-stained with MitoTracker red, DAPI and the merged images. The dotted square inset indicates the region of magnification showed in panel (**C**). (**D**) Scatter plot shows corrected total cell fluorescence for MM96L and NFF cells treated with 200 µg/mL Octpep-1 or vehicle (DMSO 0.1%). Corrected Total Cell Fluorescence = ((Cell Area × Mean Cellular Fluorescence)-(Cell Area × Mean Background fluorescence)). Octpep-1 accumulates in the perinuclear area of MM96L cells with some spots resembling vesicles (lysosomes or late/recycling endosomes), suggesting certain compartmentalization. Overlapped fluorescence for DAPI and FITC-Octpep-1 from representative (**E**) NFF and (**F**) MM96L cells. Data are expressed as mean ± SEM, vehicle (*n* = 50–90 cells) and Octpep-1 (*n* = 90–120 cells), 5–6 coverslips/treatment from 3 independent experiments. Data are expressed as mean ± SEM, ** *p* < 0.01, *** *p* < 0.001.

Our current unpublished data show that Octpep-1 possesses cytotoxic and selective properties for melanoma cells over fibroblasts (Moral-Sanz et al., unpublished data). In order to identify and characterize the molecular mechanisms underlying the biological activity of any bioactive peptide, it is required to determine whether the peptide triggers specific signaling cascades from the cell surface or intracellular organelles. Accordingly, we first studied the capacity of Octpep-1 tagged to fluorescein (48 h incubation, 200 μg/mL) to cross the plasma membrane in MM96L cells and neonatal foreskin fibroblasts (NFF). As expected, the corrected total emitted cell fluorescence (Figure 1D) was significantly higher in cells treated with fluorescein-Octpep-1 than in cells treated with vehicle (DMSO 0.1%), ruling out any autofluorescence artifact. Intriguingly, Octpep-1 showed higher accumulation in the perinuclear area of MM96L cells (Figure 1A,D) while it was homogeneously distributed within the non-transformed NFF cells (Figure 1B,D).

### 2.2. Octpep-1 Deregulates Proteins of mTOR, Actin Cytoskeleton and EIF2 Signaling Pathways

**Figure 2:**
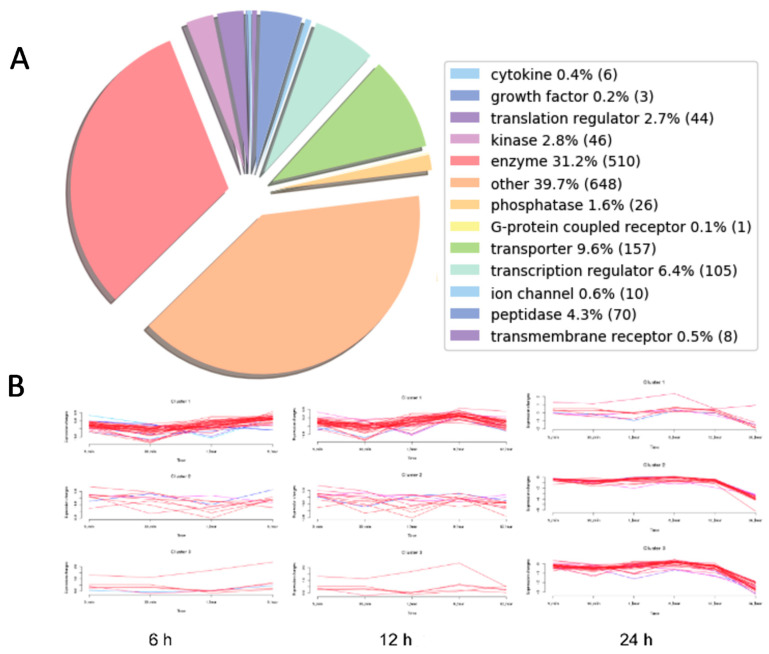
(**A**) A pie chart classifying the affected players in MM96L cells treated with 200 μg/mL of Octpep-1 during a time-course. (**B**) Ingenuity Pathway Analysis showed the pattern of three distinct clusters at 6, 12 and 24 h in MM96L cells treated with 200 μg/mL.

**Figure 3:**
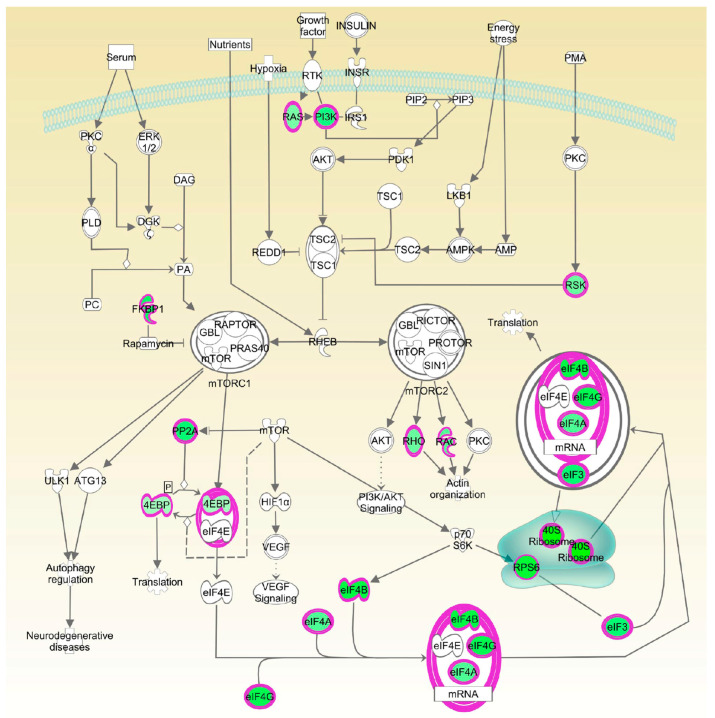
Ingenuity Pathway Analysis showing the downregulated proteins (green colour) in mTOR pathway due to Octpep-1 treatment in melanoma cells.

We performed Sequential Windowed Acquisition of All Theoretical Fragment Ion Mass Spectra method (SWATH) analysis to unravel the affected cell signaling pathways during a time course of Octpep-1 treatment (5 min to 48 h) in MM96L melanoma cells. Sequential Window Acquisition of All Theoretical Mass Spectra (SWATH-MS) is a proteomic technique for the rapid and label free quantification of proteins in complex biological mixtures [16]. Using a pre-calculated library of spectral signatures, we were able to quantitatively measure relative expression levels of proteins under a variety of experimental conditions without the need to label or process proteins prior to analysis. SWATH analysis showed that enzymes and undetermined factors were mainly affected by the Octpep-1 treatment during the time course (Figure 2A) in comparison to vehicle control (DMSO). Ingenuity Pathway Analysis (IPA) showed the pattern of three distinct clusters at 6, 12, and 24 h, respectively (Figure 2B). In addition, IPA analysis revealed that actin cytoskeleton, PI3K/mTOR signaling, and EIF2 function were the most consistently deregulated pathways in the treated melanoma cells. The affected proteins of PI3K/mTOR pathway are shown (Figure 3). We observed that granzyme A and thioredoxin signaling pathways were initially up-regulated at 5 and 30 min. EIF2 signaling proteins were enhanced after 30 min of treatment and maintained so for 24 h. Additionally, proteins regulating actin cytoskeleton were up-regulated at 1 h and up to 12 h after being exposed to Octpep-1.

### 2.3. Octpep-1 Inhibits the PI3K/AKT Pathway Activated by Insulin in Melanoma Cells

**Figure 4:**
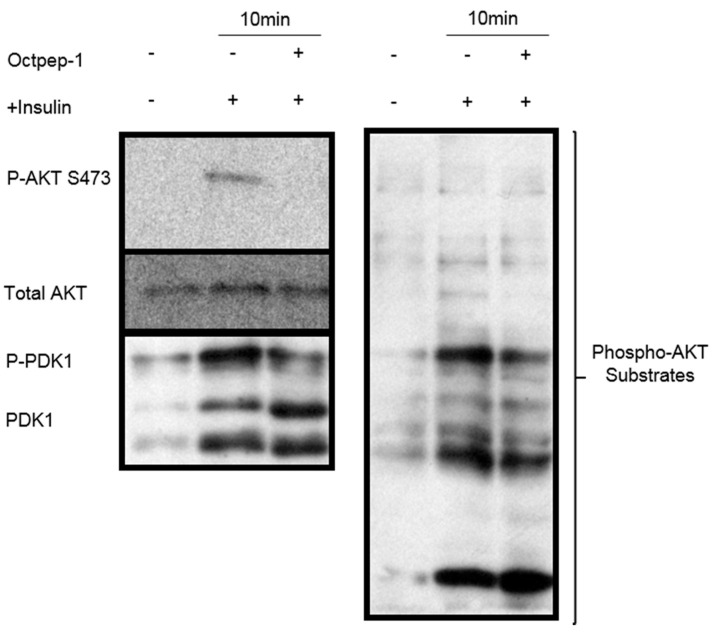
(**A**) Octpep-1 after 10 min of treatment inhibited P-AKTS473 and P-PDK1 as well as (**B**) various P-AKT substrates in insulin activated MM96L cells.

We examined the molecular signaling cascades underlying the anti-proliferative profile of octopus-treated MM96L in the PI3K/AKT pathway, which has been described to mediate proliferation in melanoma cells [17]. Following insulin stimulation for 10 min, we observed that Octpep-1 prevents the phosphorylation of AKT at residue Ser473 in MM96L cells (Figure 4A). Similarly, Octpep-1 also inhibited the phosphorylation of PDK1, which is essential for PDK1 localization at the plasma membrane and AKT phosphoylation (Figure 4A) [18]. Accordingly, we observed that Octpep-1 induces a significant reduction of phosphorylated AKT-substrates (Figure 4B).

### 2.4. Time-Course Genome-Wide Gene Expression Revealed the Alteration of Various Metabolic, Immunological or Cancer-Related Pathways in Melanoma Cells Treated with Octpep-1

**Figure 5:**
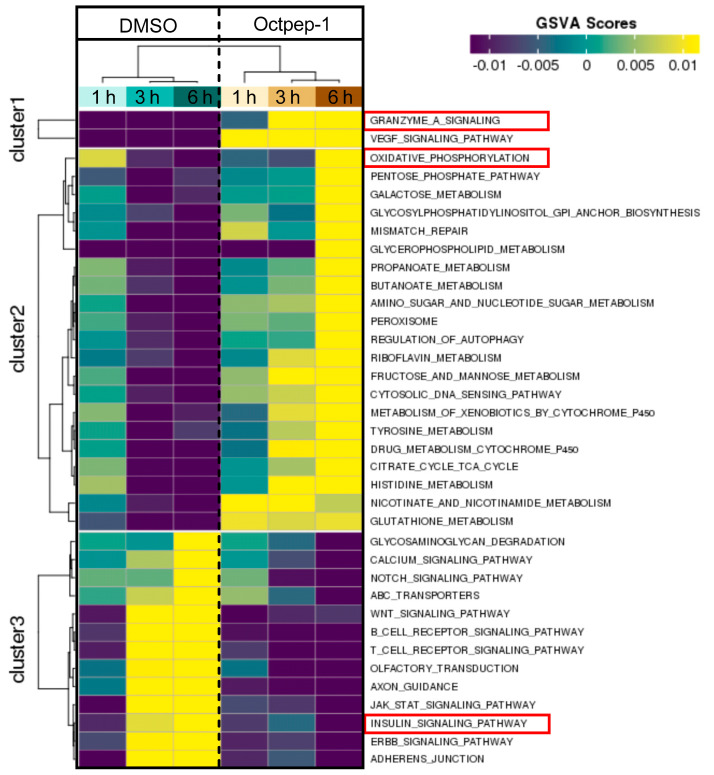
Gene-set enrichment analysis (GSVA) revealed the affected signaling pathways due to Octpep-1 or DMSO (vehicle)-treated MM96L cells. Red boxes highlight some of the pathways identified also by proteomics.

**Figure 6:**
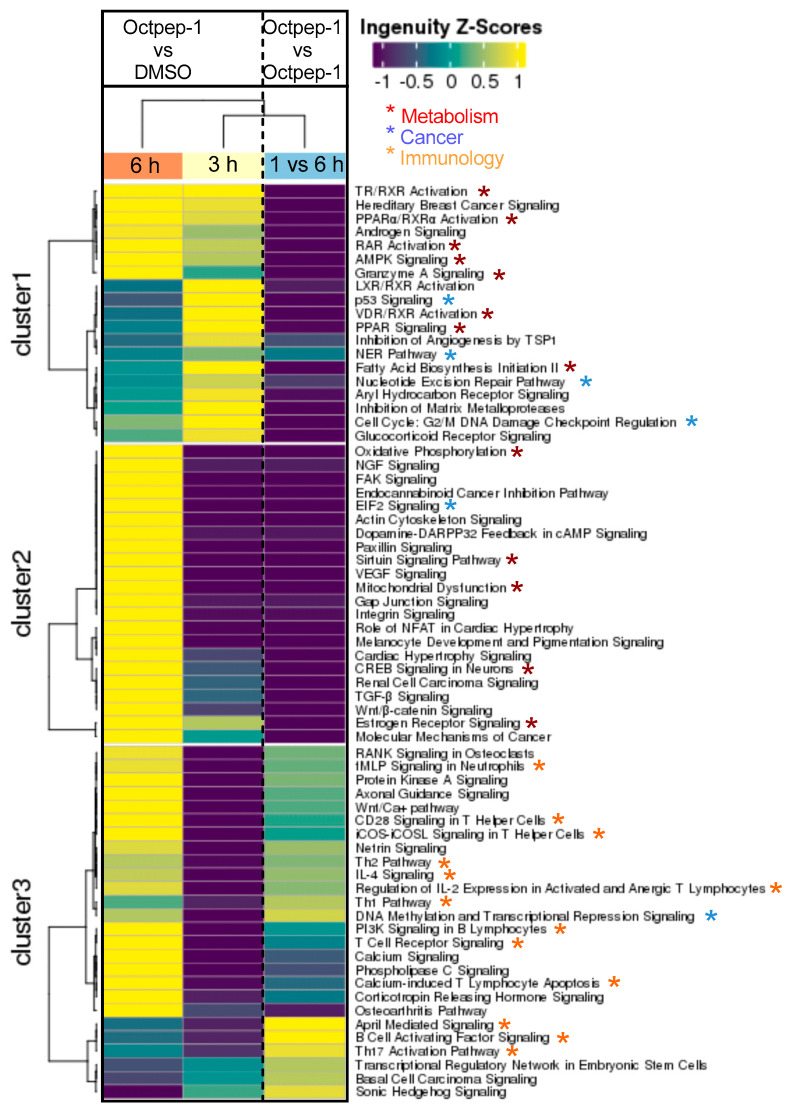
Ingenuity Z score analysis showcased that metabolism, cancer, and immunology signaling pathways were mainly downregulated at 1, 3, or 6 h after Octpep-1 treatment in melanoma cells.

To complement the proteomics output, we performed genome-wide gene expression analysis using RNAseq at 1, 3, and 6 h after Octpep-1 treatment with 200 µg/mL in MM96L cells. Like in our proteomics study, granzyme A—a pathway that is involved in cell death independent of caspase signaling—was similarly flagged and upregulated at 3 h and 6 h. Similarly, oxidative phosphorylation (OXPHOS) and insulin signaling stood out among the common affected pathways present in both omic experiments (Figure 5). Interestingly, oxidative phosphorylation was inhibited at 1 h and 3 h but was up-regulated at 6 h of Octpep-1 treatment. The RNAseq analysis showed various metabolic, tumorogenic and immunological pathways affected in MM96L cells treated with 200 μg/mL Octpep-1 at 30 min, 1, 3, and 6 h (Figure 6). More specifically, fatty acid biosynthesis initiation was one of the most deregulated pathways after 3 h of Octpep-1 treatment. Of note was also the deregulation imposed on mitochondrial dysfunction signaling during the time-course exposure (1–6 h) to Octpep-1. It is striking that in these pathways, there were 21 significantly deregulated genes. A similar pattern was recorded for oxidative phosphorylation and estrogen receptor signaling pathways with 19 and 18 deregulated genes, respectively.

### 2.5. The Combination of Octpep-1 with Rapamycin or LY3214996 Synergestically Inhibits the Proliferation of BRAF-Melanoma

**Figure 7:**
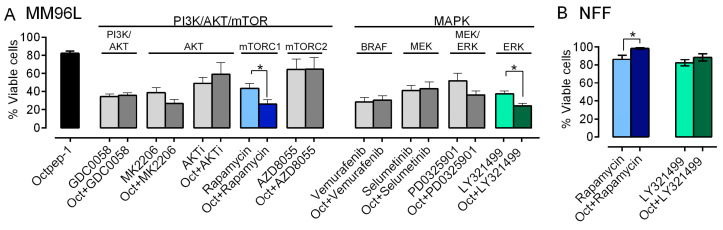
Screening of Octpep-1 and several PI3K/AKT/mTOR and MAPK inhibitors alone or in combination in MM96L cells. (**A**) The combination of Octpep-1 with LY321499 or rapamycin enhanced the antiproliferative capacity of the former in MM96L and (**B**) seemed innocuous in NFF cells. The indicated inhibitors were used at the following concentrations: 4 µM GDC0058, 0.7 µM MK2206, 1.25 µM AKTi, 5 µM rapamycin, 2.5 µM AZD8055, 2 µM vemurafenib, 1 µM selumetinib, 13 µM PD0325901 and 2 µM LY321499. Data are shown as mean values ± SEM of triplicate samples and are the result of three minimum independent experiments. * *p* ≤ 0.05 is considered as statistically significant.

We screened various inhibitors of the PI3K/AKT/mTOR and MAPK signaling at concentrations that reduced the viability of MM96L cells by approximately 50% (Figure 7A). In that regard, the PI3K/AKT/mTOR signaling was blocked by 4 µM GDC0058, which inhibits PI3K/AKT; 0.7 µM MK2206, or 1.25 µM AKT inhibitors IX (InhX) (AKTi) block AKT; 5 µM rapamycin and 2.5 µM AZD8055 target mTORC1 and mTORC2, respectively. Additionally, we prevented the activation of the MAPK cascade with 2 µM vemurafenib (BRAFi), 1 µM selumetinib that inhibits MEK, 13 µM PD0325901 blocks MEK/ERK and 2 µM LY321499 as an ERKi. Octpep-1 at 100 μg/mL reduced the viability of MM96L cells by approximately 20%. Parallel experiments demonstrated a dose-dependency in the effects of Octpep-1, where 200 μg/mL Octpep-1 alone showed a modest 40% reduction in the viability of MM96L with no significant effects in the viability of NFF cells (Moral-Sanz et al., Unpublished data). This prompted us to examine whether Octpep-1 acts synergistically with other antitumor FDA-approved drugs and augments its cytotoxicity. Indeed, we found that the combination of Octpep-1 with the mTORC1 inhibitor rapamycin or the ERK/MAPK-inhibitor LY3214996 significantly potentiated the antiproliferative properties of our candidate in MM96L as compared to Octpep-1 or the inhibitors alone (Figure 7A). Most importantly, both combinations had minimum effect on the viability of NFF cells (Figure 7B). Indeed, the combination of Octpep-1 with rapamycin significantly reversed the cell toxicity caused by Octpep-1 or rapamycin alone. The synergy of Octpep-1 with targeted treatments against mTORC1 and ERK signaling highlights the benefit of combinatorial therapies and the potential of Octpep-1 as an excellent candidate to safely potentiate antitumoral therapies in BRAF-mutated melanoma.

### 2.6. Octpep-1 Combined with LY3214996 Compromises the Mitochondrial Respiration, Non-Glycolytic Acidification and Glycolytic Capacity in MM96L Cells

**Figure 8:**
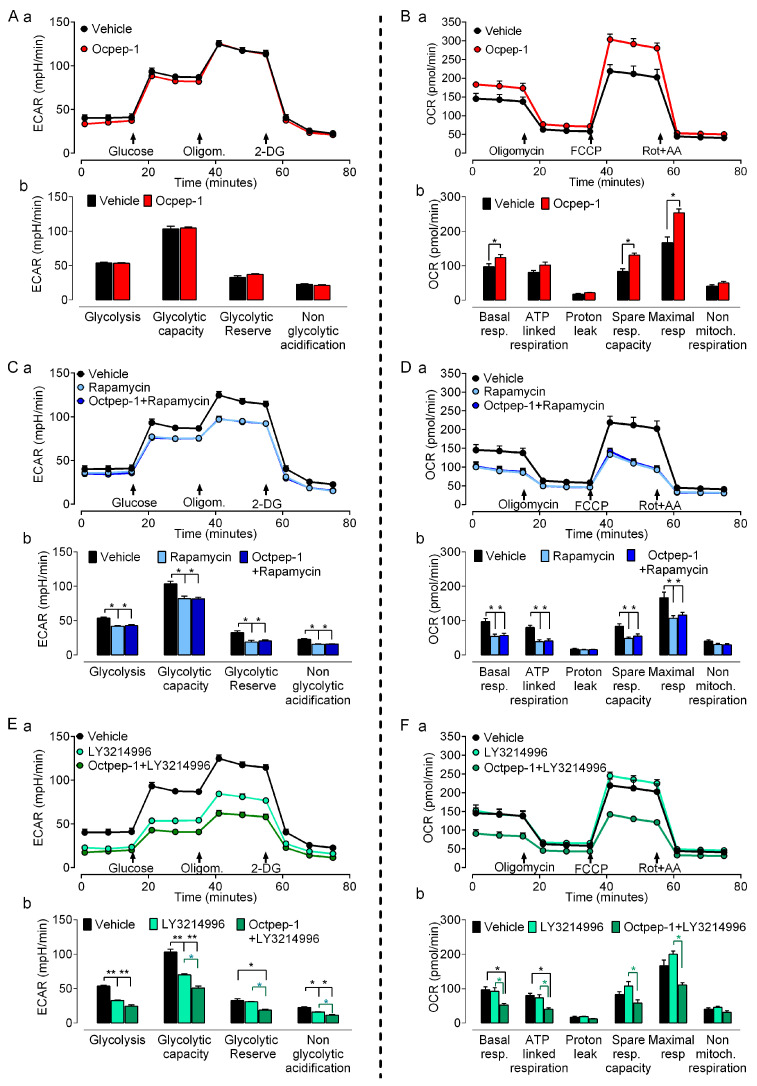
Seahorse analysis for extracellular-acidification rates (ECAR, left) and oxygen-consumption rates (OCR, right) in MM96L cells treated for 24 h with vehicle (DMSO 0.1%) or 200 µg/mL Octpep-1 (**A**,**B**), 5 µM rapamycin or Octpep-1 +5 µM rapamycin 1 (**C**,**D**) and 2 µM LY3214996 or Octpep-1 +2 µM LY3214996 (**E**,**F**). Traces from Seahorse assays (**a**) show real time measurements with arrows signaling addition of inhibitors to interrogate the respiratory and glycolytic parameters summarized in the bar charts (**b**). Data indicate means ± SEM for *n* = 4–6 independent experiments, * *p* < 0.05 and ** *p* < 0.01.

RNAseq studies implied that Octpep-1 alters OXPHOS and therefore mitochondrial function in MM96L cells. In addition, the enhancement of the antiproliferative activity of Octpep-1 in combination with rapamycin or LY3214996, which inhibits pathways directly involved in nutrient availability including carbohydrates, suggested that Octpep-1 may also target glycolysis. Next, we investigated whether these effects would translate into quantitative changes in mitochondrial respiration and glycolysis (Figure 8). This was assessed by a Seahorse Flux analyzer, which measures oxygen consumption rate (OCR), a measure of OXPHOS, and extracellular acidification rate (ECAR), a measure of lactate production by glycolysis. To investigate the glycolytic flux, we used sequential injections of glucose (10 mM) to evaluate basal glycolysis, the ATP synthase inhibitor oligomycin (3 μM) to evaluate glycolytic capacity and the hexokinase inhibitor 2-deoxy-glucose (2-DG, 50 mM) to evaluate glycolytic reserve. To examine mitochondrial respiration, we first determined basal oxygen consumption, followed by sequential injections of the ATP synthase inhibitor oligomycin (2 μM), the uncoupler fluoro-carbonyl cyanide phenyl-hydrazone (FCCP, 0.6 μM), and the combination of complex I and III inhibitors, rotenone and antimycin A (0.5 μM) that allow the estimation of spare capacity. Octpep-1 did not affect glycolysis by itself (Figure 8A) and did not act synergistically with the inhibition of mTORC1 rapamycin (Figure 8B). However, Octpep-1 enhanced the inhibitory capacity of the ERK inhibitor LY3214996 to reduce the glycolytic flux (Figure 8E). This included significant reductions in the non-glycolytic acidification and the glycolytic capacity and reserve that the ERK inhibitor was not initially affecting (Figure 8E). Octpep-1 was found to increase the basal and maximal mitochondrial respiration as well as the spare respiration capacity (Figure 8B), effects that were independently prevented by rapamycin (Figure 8D) and LY3214996 (Figure 8F). Interestingly, the combination of Octpep-1 with LY3214996 did not impair mitochondrial respiration like Octpep-1 does alone, translated to a significant reduction of the basal and maximal levels.

Similar changes in OXPHOS have been previously described in melanoma cells as part of the metabolic rewiring following targeted therapy [19,20,21]. These were associated with ROS production due to an inefficient OXPHOS [20,21] and pointed to a therapeutic strategy for intervention [22]. This potentiation shows the characteristics of a metabolic catastrophe [23] in which compromised mitochondrial respiration and low ATP coupling, meet further reductions in the non-glycolytic acidification, glycolytic capacity and reserve (Figure 8E,F).

## 3. Discussion

We observed by fluorescence microscopy that Octpep-1 accumulates in MM96L cells to exert its biological activities and this was confirmed by both proteomics and genome-wide gene expression analysis experiments. Proteomics analysis in combination with IPA detailed the clustering of various signaling pathways that were either rapidly or tardily deregulated after Octpep-1 treatment in melanoma cells. Interestingly, there were also pathways such as PI3K/mTOR or actin cytoskeleton that were consistently altered (30 min 24 h).

Changes in thioredoxin, granzyme A, and EIF2 signaling pathways suggested that cell survival mechanisms were initiated early within 5 to 30 min of Octpep-1 treatment in MM96L cells. EIF2 activation is of interest since it arrests tumor progression by manipulating the metabolism and redox status [24]. In addition, the granzyme signaling A pathway activation leads to cell death via caspase-independent pathways [25]. This was in accordance with lack of cleavage and activation of caspase 3 levels and intact mitochondrial membrane potential after 24 h of octopus peptide exposure (Moral-Sanz et al., unpublished data). After 1h we observed an activation of actin cytoskeleton and VEGF signaling proteins, which are linked to crosstalk and cell migration and thus, to metastasis. Indeed, actin cytoskeleton remained upregulated after 12 h. At this time point, we also noted an enhancement of EIF2 and integrin pathways that most likely contributed to an orchestrated signal transduction cascade related to cell proliferation and metabolism.

Moreover, the eukaryotic initiation factor-4E (Eif4e)-binding proteins (4E-BPs) are key mTORC1 substrates that control cell proliferation and survival [26]. The mTOR pathway is located downstream of AKT signaling, with the latter shown to be inhibited by Octpep-1. EIF4B proteins downstream of mTORC1 were also inhibited. This family of proteins leads to the translation of oncogenes and are directly related to tumorigenesis [27]. Thus, its inhibition most likely prevents proliferation in melanoma cells. Interestingly, upregulated levels of 4E-BPs, which are cancer repressor proteins and mTOR major substrates for phosphorylation [28] warrant further studies to elucidate a concise description of the mode of action of Octpep-1 alone or in combination with other melanoma therapies.

The proteomics data were supported and complimented further by RNAseq. The fingerprint of Octpep-1 in MM96L cells was grafted by its interactions with granzyme A signaling, VEGF, EIF2, oxidative phosphorylation, insulin signaling, actin cytoskeleton and MMP2 proteins, among others. In addition, RNAseq analysis showcased the downregulation of the ERbB pathway, whose downstream regulators compose the PI3K/AKT/mTOR pathway. We also observed many other metabolic, immunology or cancer-related deregulated signaling pathways, highlighting the wide biological action of Octpep-1 that needs to be unveiled and mapped in melanoma cells.

Understanding the mode of action of Octpep-1 is essential to cultivate its therapeutic potential as a drug candidate against melanoma of BRAF-mutation. In the clinic, the rapid antitumoral responses observed with monotherapy inhibitors (BRAFi or MEKi) are undermined due to the development of acquired resistance followed by tumor progression [3]. This establishes a hallmark in the treatment of advanced or metastatic BRAF-melanoma, which is mainly associated with a paradoxical activation of MAPK [3,4]. Hence to circumvent this issue, combinatory approaches are deemed promising. The omics evaluation of our candidate showed that Octpep-1 inhibits the ERbB/PI3K/AKT/mTOR pathway. This prompted us to examine its antiproliferative properties in combination with other inhibitors in melanoma BRAF (V600E) cells. We have characterized in detail the antiproliferative profile of Octpep-1 in melanoma BRAF-mutated cells and have shown that it reduces the tumor progression in nude xenograft mice (Moral-Sanz et al., unpublished data). However, Octpep-1 is active at relatively high concentrations (200 μg/mL in cultured cells vs. 60 mg/kg in mice) (Moral-Sanz et al., unpublished data). Consequently, we examined whether its combination with other inhibitors would be a more effective therapeutic approach against BRAF (V600E) melanoma cells. Indeed, our study revealed specific synergistic antitumoral activities of Octpep-1 in combination with rapamycin (mTOR inhibitor of complex 1) or LY3214996 (EKR1/2i). In both combinations the viability of the melanoma cells was reduced to approximately 24% instead of ≈40% for the inhibitors (rapamycin 5 μM and ERKi 2 μM) and ≈80% for the Octpep-1 (100 μg/mL) alone. Proteomics revealed that 4EBPs that are located downstream of mTORC1 were inhibited. Hence, the blockage of mTORC1 by rapamycin strengthens the effectiveness of our candidate in the blockage of the ERbB/PI3K/AKT/mTOR pathway and potentially arrests the initiation of a drug resistance mechanism in melanoma cells. In addition, the synergistically abolishing effect of Octpep-1 with the ERKi is in accordance with the current literature stating that MAPK is stimulated in BRAF melanoma [3,4].

There is an interplay and an extensive crosstalk between MAPK and PI3K/AKT pathways. MAPK resistance to BRAF inhibitors can be mediated through the enhancement of the latter signaling pathway [29]. In fact, cross-activation of the PI3K/AKT/mTOR pathway is mediated by the activation of ERK or RSK and consequently mTORC1 [30]. Furthermore, AKT inhibition reverses the acquired drug resistance in combination therapy with dabrafenib and trametinib in vitro [29]. Synergistic effects and delayed acquisition of resistance have also been shown with the combination of vemurafenib (BRAFi) and SCH722984 (ERKi) in BRAF mutant melanoma cells [30]. Therefore, it might be a favorable regime to combine a BRAF inhibitor with an inhibitor of the PI3K-AKT like Octpep-1 or MAPK pathway in BRAF melanoma patients.

Efficacy of anticancer agents is not solely dependent upon proliferative index. Tumors use diverse mechanisms to thrive and this influences drug effectiveness [31]. Targeting tumor cell metabolism may either promote or prevent progression and thus could be an attractive anti-tumoral therapeutic approach. In addition, the fact that omics identified various metabolic-related signaling pathways, all together, prompted us to examine the effect of Octpep-1 in various metabolic parameters in melanoma BRAF-mutated cells. The combination of Octpep-1 with the ERK inhibitor coordinated parallel reductions in glycolytic and mitochondrial fluxes that compromised the metabolic flexibility of MM96L cells. Therefore, this challenged metabolism set unfavorable conditions for growth that supports the potential of Octpep-1 to reduce the viability of melanoma cells.

## 4. Conclusions

In this study we shed light onto the therapeutic perspective of a small linear octopus peptide as a potential drug candidate targeting BRAF (V600E) melanoma. We showed that Octpep-1 is a PI3K/AKT/mTOR inhibitor that acts synergistically with rapamycin or ERK inhibitor to enhance its antiproliferative profile. In fact, the combination of Octpep-1 with the ERK inhibitor orchestrated many metabolic changes in the melanoma treated cells to enhance the antiproliferative capacity of the octopus peptide. Equally important are our observations that these combinations did not interfere with the proliferation of the healthy NFF cells. These results warrant further studies, including to evaluate in vivo these approaches to be able to draw final conclusions on the therapeutic potential of Octpep-1 in combination with rapamycin or LY3214996 in melanoma patients.

## 5. Materials and Methods

### 5.1. Reagents

All media for cell culture, was purchased from Invitrogen/Gibco ThermoFisher, Massachusetts, USA. MitoTEMPO was from Cayman Chemicals (Michigan, USA) (# 16621). Antibodies were obtained from Cell Signaling Technology (Danvers, MA, USA) including: Rabbit anti-Phospho AKT^Ser473^ (#4060), Total AKT (#4691), Phospho-PDK1 Anti-rabbit HRP (#A0545) was from Sigma-Aldrich (Munich, Germany) etc.

### 5.2. General

All reagents related to chemical synthesis of the compounds were obtained commercially and were used without further purification. Peptides were dissolved in water and mixed 1:1 (*v*/*v*) with *α*-cyano-4-hydroxycinnamic acid matrix (7 mg/mL in 50% MeCN, 5% formic acid) and mass spectra acquired in positive reflector mode. All reported masses are for the monoisotopic [M + H]^+^ ions. Amino acids were purchased from IRIS Biotech GmbH (Marktredwitz, Germany), Bachem (Bubendorf, Switzerland), or ChemImpex Inc. (Wood Dale, IL, USA). Fmoc-Met-Wang resin was obtained from Peptide International (Louisville, KY, USA).

Eluents for RP-HPLC consisted of 0.05% trifluoroacetic acid (TFA)/H_2_O (solvent A) and 90% ACN/0.045% TFA/H_2_O (solvent B). Analytical HPLC quality control analysis of the investigated peptide was performed on a Shimadzu LC20AT system using an Agilent Eclipse Plus C18, 2.1 × 50 mm, 5 μm column with a flow rate of 0.3 mL/min at 45 °C. A gradient of 10 to 50% B over 40 min was used, with detection at 214 nm.

Preparative HPLC were performed on a Waters 600E system using a gradient of 0 to 50% B over 50 min. For the prep. HPLC of the crude peptide an Agilent Eclipse XBD-C18, 21.2 × 250 mm, 5 µM column with a flow rate of 16 mL/min was used. Monoisotopic molecular masses were determined by MALDI-MS on a 4700 Proteomics Analyzer (Applied Biosystems, Waltham, MA, USA) mass spectrometer in positive ion mode.

### 5.3. Chemical Synthesis of Octpep-1 and Labeled Peptide

**Figure A1:**
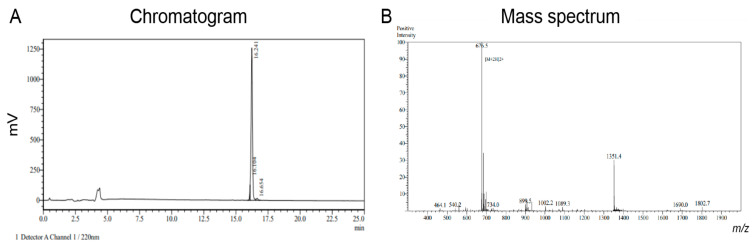
(**A**) Rp-HPLC and (**B**) mass spectrum of Octpep-1 showing its purity. Theoretical reported MW was 1351.44 and observed MW was 1351.0.

Octpep-1 (H-DPPSDDEFVSLM-OH) was assembled on a 0.25-mmol scale on a Fmoc-Met Wang resin following the Fmoc/tBu-SPPS protocol. Couplings were performed in dimethylformamide (DMF) using 4 equivalents of Fmoc-amino acid/*O*-(6-Chlorobenzotriazol-1-yl)-N, N, N’, N’-tetramethyluronium hexafluorophosphate (HCTU)/DIPEA (1:1:1.1) relative to resin loading for 15 min. Fmoc deprotection was achieved using 50% piperidine/DMF (2 × 1 min). Final cleavage and global side chain deprotection were accomplished by stirring in 90% TFA, 5% TIPS, and 5% H_2_O for 90 min at ambient temperature. The suspension was filtered, washed with TFA, the filtrate concentrated under steady nitrogen flow to a minimal amount, and the peptide precipitated and washed with cold diethyl ether. The precipitate was filtered off and then dissolved in 0.05% TFA in 50% MeCN/H_2_O and lyophilized. The crude peptide was purified by preparative HPLC and the mass analyzed by MS in positive ion mode using an ABSciex API 2000TM. Pure fractions were combined and lyophilized (6.5 mg) (Figure A1).

**Figure A2:**
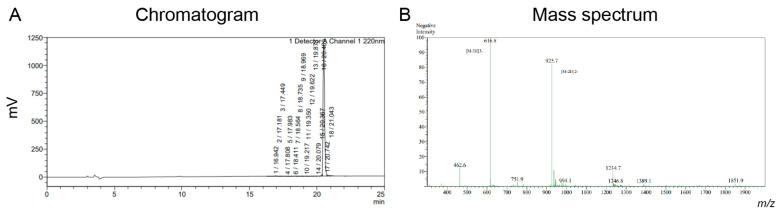
(**A**) RP-HPLC and (**B**) mass spectrum of labeled Octpep-1 showing its purity. Theoretical reported MW was 1853.98 and observed MW was 1853.4.

DPPSDDEFVSLM-OH with a modification at N-T:FITC-Ahx was chemically synthesized using FMOC-SPPS and purchased by GenScript. Pure fractions were lyophilized (20 mg) (Figure A2). In brief, Octpep-1 was tagged with fluorescein isothiocyanate (FITC) and using a spacer, 1,6-aminohexanoic acid (Ahx). For efficient N-terminal labeling of peptides by solid phase synthesis, a spacer is recommended between the fluorophore (fluoroscein) and the N-terminus of the peptide [32].

### 5.4. Cell Culture

The MM96L, human melanoma and patient-derived cell line as well as the non-transformed neonatal foreskin fibroblast (NFF) cells were maintained in a humidified incubator at 37 °C and 5% CO_2_. The melanoma cells were cultured in RPMI-1640 media supplemented with 10% FBS, and 2 mM Glutamax^TM^. NFF cells were grown in RPMI-1640 containing 10% FBS. In all cells, penicillin/streptomycin (PS) (100 U/mL each) was added in the media. Cells were passaged at approximately 90% confluency and experiments were performed with passages up to 10. All cell lines were mycoplasma free.

### 5.5. Fluorescence Microscopy

Octpep-1 tagged with fluorescein at the N-terminus was purchased from GenScript (Leiden, The Netherlands). MM96L cells were grown in coverslips and treated with 200 µg/mL Octpep-1 tagged with fluorescein at 37 °C for 48 h. Additionally, mitochondrial co-staining was performed by incubation with 100 nM MitoTracker Red (excitation, 581 nm; emission, 644 nm) for 30 min at 37 °C followed by washes with PBS and 30 min fixation with 4% PFA at room temperature. Fluoroshield Mounting Medium with DAPI (4′,6-diamidino-2-phenylindole) was used to stain the nucleus and preserve fluorescence in the fixed samples. Images of the emitted fluorescence were acquired at 300, 50, and 100 ms of exposure for green, red, and blue fluorescence, respectively, using a Leica DMIL microscope with a Plan- Apochromat 63 × 1.4 NA oil immersion objective. Analysis was completed using ImageJ-Fiji software (National Institutes of Health, Tennessee, USA). Data are expressed as corrected total cell fluorescence using the following equation [(Cell Area × Mean Cellular Fluorescence) − (Cell Area × Mean Background fluorescence)].

### 5.6. Cell Lysis of Stimulated and Unstimulated Melanoma Cell Line for Protein Library Generation and SWATH Quantitation

For the protein library generation approximately 30 million near confluent unstimulated adherent MM96L melanoma cells were trypsinised for 2 min and then harvested followed by three washes with Dulbecco’s PBS (Gibco, Waltham, MA, USA) with centrifugation at 800× *g* for 5 min between each wash. For SWATH quantification, 100,000 adherent mm96l melanoma cells were harvested in triplicate at different time points post-stimulation (unstimulated, 5 min, 30 min, 1, 6, 12, and 24 h) and were washed three times with Dulbecco’s PBS (Gibco, Waltham, MA, USA) with centrifugation at 800× *g* for 5 min between each wash. Stimulated and unstimulated MM96L cells were lysed on ice by resuspending the cells pellet in 100 mM TEAB (Thriethylammonium bicarbonate), 1% SDS (sodium dodecyl sulfate), 10 mM CHAPS, 5 mM MgCl_2_ supplemented with 1× Roche Complete protease inhibitors. DNA and RNA were then degraded by the addition of 50 µL and 2 µL, respectively, of 50 mM Tris, 20 mM NaCl, and 2 mM MgCl_2_ at 1 unit/µl of ultrapure Benzonase (Sigma, Munich, Germany) followed by incubation at 4 °C for 45 min with constant agitation. Protein concentration was determined using the Pierce BCA Assay (ThermoFisher Scientific, Waltham, MA, USA) following the manufacturer’s protocol.

### 5.7. Peptide Fractionation by OFFGEL^TM^ Electrophoresis (OGE) for Protein Library

600 µg of proteins in lysis buffer from the unstimulated MM96L cells were aliquoted and reduced with 0.5 M DTT stock solution added to a final concentration of 20 mM. Samples were then incubated at 75 °C for 10 min and cooled at room temperature for 10 min. Proteins were then alkylated for 30 min in darkness with 0.5 M iodoacetamide (IAA) stock solution to a final concentration of 40 mM. A 10 kDa molecular weight cut-off filter tube (Amicon Ultra-4, Millipore, Burlington, MA, USA) was equilibrated by spinning 700 µL of 100 mM TEAB through the filter at 3100× *g* for 20 min. The protein sample was mixed with 6 volume of 8 M Urea in 100 mM TEAB, 10% isopropanol, and transferred to the filter unit and spun at 3100× *g* for 40 min in a 5810R (Eppendorf, Hamburg, Germany) centrifuge. Detergent removal by buffer exchange was performed using a first wash of 500 µL of 8 M Urea in 100 mM TEAB, 10% (*v*/*v*) isopropanol followed by a wash with 500 µL of 100 mM TEAB, 10% isopropanol then two successive washes with 500 µL of 50 mM TEAB at 3100× *g* for 40 min each. The concentrated protein solution was then collected from the filter unit and transferred to an Eppendorf microcentrifuge tube and protein digestion was performed by adding Tryspin enzyme in 50 mM TEAB in an enzyme to protein ratio of 1:50 then mixed well before incubation at 37 °C in a wet chamber overnight. Peptides from the digested protein sample were desalted using a Sep-Pak Vac C18 cartridge (Waters, Milford, MA, USA) and the eluted fraction was lyophilized with a speed vacuum prior to OFFGEL^TM^.

The 3100 OFFGEL Fractionator and OFFGEL kit pH 3-10 (Agilent Technologies, Waldbronn, Germany) with a 24-well setup were prepared according to the manufacturer’s protocols. Lyophilized peptide mixtures were resuspended to a final volume of 3.6 mL using the OFFGEL peptide sample solution. IPG gel strips (24 cm) with a 3–10 linear pH range (GE Healthcare, Munich, Germany) were rehydrated with the Peptide IPG Strip Rehydration Solution according to the manufacturer’s protocol and 150 µL of sample was loaded in each well. Peptides were isoelectrically focused with a maximum current of 50 µA until 50 kV-h were achieved. Twenty-four fractions were recovered from each well and the wells were rinsed with 150 µL of water/methanol/formic acid (49/50/1) for 15 min. Each fraction was lyophilized and then resuspended in 30 µL of H_2_O with 2% ACN (acetonitrile), 0.1% formic acid (*v*/*v*) prior to LC-MS/MS analysis.

### 5.8. Protein Preparation for SWATH Quantitation

The different protein samples in triplicate for each time point were prepared according to the modified FASP protocol for high throughput proteomics [33]. Briefly, 50 µg of proteins in lysis buffer from each of the time point replicates were aliquoted and reduced by the addition of 0.5 M DTT stock solution to a final concentration of 20 mM followed by incubation at 75 °C for 10 min and cooling at rt for 10 min. Protein were then alkylated for 30 min in darkness with 0.5 M IAA stock solution to a final concentration of 40 mM. Eight volumes of 8 M Urea, 10% isopropanol in 100 mM TEAB was then added to the protein samples. A 30 kDa molecular weight cut-off filter plate (Acroprep advance 96-well Omega filter plates, PALL, New York, NY, USA), coupled to a deep U-bottom well plate (Axygen, Tewksbury, MA, USA) for collection, was equilibrated by briefly spinning 200 µL of 60% isopropanol through the filter at 3100× *g*. Protein samples were then transferred to different wells on the plate and spun at 3100× *g* for 30 min in a 5810R (Eppendorf, Hamburg, Germany) centrifuge with adapted plate bucket. Detergent removal by buffer exchange was performed in two successive washes with 8 M urea, 10% isopropanol in 100 mM TEAB with centrifugation at 3100× *g* for 30 min between each wash. Urea was then removed by two washes with 10% isopropanol in 50 mM TEAB with centrifugation at 3100× *g* for 30 min between each wash. A final wash with 50 mM TEAB was performed with centrifugation as described above. Protein digestion was then performed by adding 1 µg of trypsin in 50 mM TEAB to the wells and incubating overnight at 37 °C. Peptides were recovered over the deep V-bottom plate (Axygen, Life Sciences, Arizona, USA) using an initial spin at 3100× *g* for 10 min followed by two centrifugations with 50 µL of 50 mM TEAB. Recovered peptides were dried in a speed vacuum for 4 h at 45 °C. Dried peptides were resolubilized in H_2_O, 0.1% TFA (*v*/*v*), desalted and normalized to 5 µg using ZipTip^TM^ C18 tips (Millipore, Massachusetts, USA) according to manufacturer’s protocol. Eluted peptides were lyophilized and then resuspended in 30 µL of H_2_O with 2% ACN (acetonitrile), 0.1% formic acid (*v*/*v*) spiked with the iRT calibrant (Biognosys, Zurich, Switzerland) prior to LC-MS/MS analysis.

### 5.9. Protein Identification/Quantification Using LC-MS/MS

All peptide samples were chromatographically separated by a 10 µL injection on an Eksigent cHiPLCTM-nanoflex system using a 15 cm long chromXP C18-CL column (particle size 3 µM, 120 Å, 200 µM × 6 mm). A pre-concentration step (10 min) was performed employing a chromxp trap (C18-CL, 3 µM, 120 Å, 200 µM × 6 mm) before commencement of the gradient. A flow rate of 500 nl/min was used for all experiments. The mobile phase consisted of solvent A (0.1% formic acid [aq]) and solvent B (100 acetonitrile/0.1% formic acid [aq]) were used for the three consecutive linear gradients for peptide elution: 5–10% solvent B (acetonitrile/0.1% formic acid) over 2 min, 10–40% solvent B over 58 min and 40–50% solvent B over 5 min. A final gradient from 50% to 95% solvent B in 10 min was used to clean the column. Eluates from the RP-HPLC column were directly introduced into the NanoSpray II ionisation source of a TripleTOF 5600 MS/MS System (AB Sciex, Framingham, MA, USA) operated in positive ion electrospray mode. The data for OFFGEL fractions were obtained using Data Dependent Acquisition (DDA) as previously described [34], while the quantification data from the different time points were obtained using the Sequential Windowed Acquisition of All Theoretical Fragment Ion Mass Spectra method (SWATH) under the same conditions as the DDA experiments and a rolling collision energy method was used to fragment all ions in a set of 26 sequential overlapping windows of 25 AMU over a mass range coverage of 350–1000 (*m*/*z*). An accumulation time of 100 ms was used for each fragment ion scan resulting in a total cycle time of 2.9 s. Data were acquired and processed using Analyst TF 1.7 software (AB SCIEX, Victoria, Australia).

### 5.10. Protein Library Generation and Bioinformatic Analysis of SWATH Protein Quantitation

Spectral searches of processed LC-MS/MS data were performed using ProteinPilot v4.5 (AB SCIEX) using the Paragon algorithm (version 4.5.0.0). Background correction was used and biological modifications specified as an ID focus. The detected protein threshold was set as 0.5 and the false-discovery rate (FDR) was calculated using searches against a decoy database comprised of reversed sequences. Searches were conducted against the UniProt human reference proteome set comprising 70,953 protein sequences (downloaded 13th February 2017). For spectral library generation and SWATH XIC peak area extraction PeakView v2.2.0 (AB SCIEX, Victoria, Australia) with the SWATH acquisition MicroApp was used with ion library parameters set to 6 peptides per protein, 6 transitions per peptides, a peptide confidence threshold of 99%, and FDR threshold to 1%. The XIC time window was set to 6 min and XIC width to 75 ppm. Retention times for all SWATH experiments were normalized using the spiked iRT calibrants. To generate the quantitation table files for ions, peptides, and proteins Marker View v1.2.1.1 (AB SCIEX, Victoria, Australia) was used and the relative area under peaks across the different experiments was normalized based on the iRT internal calibrant. Figures and statistical analysis were generated with Python and R language and graphical packages.

### 5.11. Western Blots

MM96L cells were lysed in cold RIPA buffer containing protease (Merck Pty Ltd., Kilsyth, Australia) and phosphatase (Roche Diagnostics, Castle Hill, Australia) inhibitors and stored at −20 °C. Protein concentrations were determined using a Pierce BCA Protein assay kit (Thermo Fisher Scientific, Waltham, USA). Samples were subjected to SDS-PAGE and blotted according to standard procedures. In brief, 10 μg of protein was loaded per lane. Antibodies used for Western blots are described in the Reagents section. Protein signals were visualized using enhanced chemiluminescence (Pierce™ ECL Western Blotting Substrate, Thermo Fisher Scientific, Waltham, MA, USA).

### 5.12. RNA Sequencing

Time course was performed at 30 min, 1 h, 3 h, and 6 h of MM96L cells treated with Octpep-1 at 200 μg/mL. The cells were then collected, and RNA extraction was performed by RNAsy kit (QIAGEN, Venlo, The Netherlands) according to manufacturer’s directions. The RNA samples were sent for sequencing to the Institute for Molecular Bioscience Sequencing Facility.

### 5.13. Pathway Analysis

To determine the differentially expressed set of genes for melanoma treated with the Octpep-1 versus vehicle treated cells at different time points, we performed Ingenuity Pathway Analysis (IPA) followed by Principal Component Analysis (PCA) and enriched with GSVA and Ingenuity Z score analysis.

### 5.14. Cell Viability Assay

Cell viability was evaluated by the MTT assay and measured at 570 nm absorbance on a microplate reader (BIOTEK PowerWave XS, Winooski, VT, USA) after 48 h of peptides’ treatment using an MTT assay (Sigma-Aldrich (Munich, Germany) as previously described [11,35].

### 5.15. Bioenergetics

Cellular bioenergetic measurements were performed using the Seahorse XFe96 Analyzer and XFe96 culture microplates (Agilent Technologies, Madrid, Spain) to investigate Oxygen Consumption Rates (OCR) and Extracellular Acidification Rates (ECAR) in MM96L. Measurements were performed after 24 h incubation with Octpep-1, ERK1/2i, mTORC1i and combination of the inhibitors with Octpep-1 in 15 × 103 melanoma cells/well in quintuplicates. OCR was tested in Seahorse XF base medium containing 15 mM glucose, 1 mM sodium pyruvate, and 2 mM L-glutamine (pH 7.4) and ECAR in Seahorse XF base medium containing 0.5 mM sodium pyruvate and 1 mM L-glutamine (pH 7.4).

### 5.16. Statistical Analysis

Statistical analyses were conducted using Prism version 8.0 (Graphpad Software Inc., San Diego, CA, USA). Data were checked for normality to choose the appropriate test (parametric or non-parametric test). Significance for the screening of various inhibitors and their combination with Octpep-1 was calculated using a one-way ANOVA and Sidak’s multiple comparisons test. Differences between Octpep-1 and its combination with rapamycin or ERKi were examined by unpaired two-tailed t-test and Mann Whitney test, respectively. In addition, for the seahorse metabolic experiments significance was calculated using a one-way ANOVA and Dunn’s multiple comparison test with *p*-values adjusted using Kruskal–Wallis or Mann–Whitney method. The data from MTT experiments are the result of at least three independent experiments done in triplicate or quadruplicate while seahorse data are the outcome of four to six independent experiments done at least in quadruplicate. Data are represented as mean ± SEM. Significance was defined at * *p* ≤ 0.05 or ** *p* ≤ 0.01.

## Data Availability

Not applicable.
